# Hygroscopy as an Indicator of Specific Surface Area in Polymer Materials

**DOI:** 10.3390/polym16050593

**Published:** 2024-02-21

**Authors:** Andrey V. Smagin, Nadezhda B. Sadovnikova

**Affiliations:** 1 Soil Science Department and Eurasian Center for Food Security, GSP-1, Leninskie Gory, 119991 Moscow, Russia; nsadovnik@rambler.ru; 2 Institute of Forest Science, Russian Academy of Sciences (ILAN), 21, Sovetskaya, 143030 Uspenskoe, Moscow Region, Russia

**Keywords:** polymer materials, dispersity, specific surface area, hygroscopy, water content, surface energy, starch, cellulose, acrylic hydrogels

## Abstract

Specific surface area (SSA) is an integral characteristic of the interfacial surface in poly-disperse systems, widely used for the assessment of technological properties in polymer materials and composites. Hygroscopic water content (*W*_h_) is an obligate indicator of dispersed materials prior to any analysis of their chemical composition. This study links both indicators for the purpose of the express assessment of SSA using widely available *W*_h_ data, on the example of natural (starch, cellulose) and synthetic (acrylic hydrogels) polymer materials. The standard BET analysis of SSA using water vapor desorption was chosen as a reference method. In contrast to the known empirical correlations, this study is based on the fundamental thermodynamic theory of the disjoining water pressure for the connection of the analyzed quantities. The statistical processing of the results for the new methodology and the standard BET method showed their good compliance in a wide range of SSA from 200 to 900 m^2^/g. The most important methodological conclusion is the possibility of an accurate physically based calculation of hydrophilic SSA in polymer materials using their *W*_h_ data at a known relative humidity in the laboratory.

## 1. Introduction

Specific surface area (SSA) is the basic characteristic of the interfacial surface in poly-disperse polymeric materials and composites. In contrast to the size distribution of particles, SSA is an integral indicator, which is convenient for its use in process models of interphase interactions and reactions [1]. The SSA factor controls the surface physical and chemical properties of polymer materials and composites, including the adsorption and desorption of dissolved substances, gases, and water vapors [1,2,3,4,5], surface energy and reactivity, sorption kinetics [6,7,8], wettability [5,6,7,8,9], and the interaction of the polymer matrix with fillers [10,11]. The study of SSA for nanodisperse materials and composites is especially relevant in connection with their synthesis as superabsorbents for modern technologies of water absorption and purification, carbon dioxide binding, and use in sustainable green chemistry and energy [4,6,9,12,13].

The most well-known methods for determining SSA are geometric calculations using particle size distributions or electron microscopy images (TEM, SEM, LEEM, etc.) and XR-computed tomography of the pore space [1,14,15,16], as well as the methods of the sorption of molecules with a known effective area [8,17,18]. For most polydisperse systems, the geometric calculation of SSA yields significantly smaller values compared to the adsorption method [1], and only in nanosystems do the results of both methods converge within an acceptable error of about 10% [19]. The group of adsorption methods according to the ISO 9277:2022 standard [18] for disperse or porous solids is based on the Brunauer, Emmett, and Teller (BET) theory of polymolecular adsorption [18]. This standard allows the use of different adsorbate molecules with a known cross-section area from 0.125 nm^2^ (water at 298 K) to 0.643 nm^2^ (n-Octan at 298 K), both non-polar (N_2_, CO_2_) and polar (H_2_O).

The low-temperature (77 K) adsorption of nitrogen molecules is the most common type of adsorption BET method for measuring SSA, implemented in commercial sorbtometers [2,5,17]. The standard BET method using nitrogen gives a range of SSA estimates from 0.1–10 m^2^/g for biopolymers (starch, cellulose, peat, etc.) [8,20,21] to 2–3 thousand m^2^/g for surface-modified nanocomposites, for example, graphene [5,6]. Since specific nitrogen adsorption does not take into account polar interactions, this method most often overestimates SSA for hydrophobic surfaces and underestimates it for hydrophilic ones. For many hygroscopic polymers with a hydrophilic (or amphiphilic) surface, this method gives 10–100 times lower SSA values compared to the sorption of polar water molecules, for example, for starch, where SSA by nitrogen is less than 0.3 m^2^/g, and SSA by water exceeds 250–300 m^2^/g [20,22]. Also, the standard method is laborious and requires special expensive equipment for low-temperature nitrogen adsorption. Simpler methods use the adsorption of dyes or water vapor to evaluate SSA in polymers [3,8,23]. Water vapor adsorption is often used for SSA analysis in plant biopolymers of food [24,25,26]. In these studies, the calculation of SSA from water vapor sorption isotherms is based not only on the standard BET model, but also on the GAB (Guggenheim–Anderson–de Boer) model, which is more adequate for S-shaped curves of water vapor sorption isotherms by polymeric materials [24,27]. A number of researchers emphasize the close correlation of the SSA index and the hygroscopic water content of materials, which is obvious, since the larger the surface, the higher the adsorption of water vapor from the air [28,29,30].

Hygroscopicity is the property of materials to absorb water vapor from the air. The dynamic equilibrium in the cycles of adsorption/desorption of water vapor is characterized by the hygroscopic water content (*W*_h_). This water content can be quite large and reach tens of percent in highly hygroscopic polymers and composite materials [12,13,23]. The equilibrium content of hygroscopic water is necessarily determined before any chemical analysis, if its data are normalized by the mass of the dry solid phase. The hygroscopicity of water-soluble organic polymers increases with the number of functional groups and molecular weight; for a slightly soluble organic material, the greater its solubility, the higher its hygroscopicity [31]. This indicator is especially important in atmospheric chemistry, pharmaceuticals, and materials science, where hygroscopicity strongly affects the functioning and technological properties of dispersed systems (aerosol particle size, light absorption and transmission, powder aggregation, their wettability and solubility, change in marketable weight at a violation of the sealing of packages, etc.) [30,32,33,34,35,36]. In this case, hygroscopicity is studied in a wide range of relative air humidity (water activity) to obtain experimental characteristics in the form of water vapor sorption isotherms or hygroscopic growth functions [32,37]. The processing of these experimental data using BET or GAB models or other physically based models of interfacial interactions of water and a solid-phase matrix makes it possible to estimate the hydrophilic SSA index, which takes into account the polar bonds of water molecules, in contrast to the standard BET estimate with nitrogen. It should be emphasized that ISO 9277:2022 [18] on the SSA adsorption method allows the use not only of nitrogen or dyes as adsorbates, but also polar water molecules. Therefore, the BET method using the sorption of water molecules is also standard, although less often used in polymer chemistry and materials science. The hydrophilic SSA, estimated through water sorption, is especially important for modeling water absorption and water retention by hygroscopic superabsorbent materials, predicting their technological quality and functionality.

Despite the obvious connection between hygroscopicity and SSA, only the rare empirical models mentioned above are known in this field [28,30]. Theoretical approaches to combine these indicators, as far as we know, do not exist. Therefore, this study was aimed at developing the fundamental thermodynamic dependence of SSA and hygroscopic water content to substantiate a new methodology for the express evaluation of SSA in polymeric materials. The main research tasks included the following:

- Derivation of the fundamental thermodynamic dependence of SSA and hygroscopic water content in dispersed materials;
- Experimental analysis of thermodynamic water retention curves and water vapor sorption isotherms in biopolymers (starch, cellulose) and synthetic composite water superabsorbents with an acrylic polymer matrix;
- Statistical comparison of the thermodynamic approach and the standard BET method for the SSA assessment, and substantiation of the methodology for assessing the SSA through hygroscopy in polymer materials.

The scientific novelty consisted in the development of a fundamental thermodynamic equation connecting the specific surface of a material, its hygroscopic water content, and the relative humidity of the air in a thermodynamic reservoir (laboratory room). This theoretical basis made it possible for the first time to substantiate a new methodology for assessing the SSA using hygroscopy data in dispersed materials, on the example of natural and synthetic polymers. The results may be accepted by a wide range of specialists in the field of polymer chemistry, atmospheric chemistry, materials science, pharmaceuticals, etc., where hygroscopic polymers and their surface interactions are studied in connection with the technological properties, functions, and behavior of these materials in the environment.

## 2. Materials and Methods

### 2.1. Tested Biopolymers and Synthetic Polymer Materials

The tested materials were represented by both natural biopolymers and synthetic polymers, which are superabsorbents of water. Two samples of polysaccharides, Starch ACS reagent, soluble, and Cellulose microcrystallic, extra pure, produced by Acros Organics BVBA, Geel, Belgium [38], were selected as natural biopolymer materials. Some of their properties, including hygroscopic water content, are given in Table 1. The relative air humidity in the laboratory usually varies, increasing in summer and decreasing in winter (heat supply season); therefore, Table 1 contains two values of hygroscopic water content for the summer (*W*_h_^S^) and winter (*W*_h_^W^) seasons. Along with hygroscopicity, Table 1 presents the traditional parameters of organic carbon content (*C*%) and *pH* (aq) for the tested polymers.

In addition to natural biopolymers, four synthetic polymer composites (superabsorbents) intended for soil conditioning were included in the tested materials (Table 1). The first two superabsorbents are well-known brands of composite acrylic materials: “Aquasorb” (SNF-group, [39]) and “Zeba” (UPL-group, [40]), based on polyacrylamide, acrylic acid, and starch. The other two polymer composites are represented by innovative soil conditioners synthesized at the Ural Chemical factory (Russian Federation, Perm) under the “Aquapastus” trademark using our patented technology (see “Section 6”). A mandatory hydrophilic base is an acrylic polymer matrix with a varying ratio of copolymers of acrylamide and acrylic acid salts (ammonium and sodium acrylates). This polymer base provides the formation of gel structures in pure aqueous and slightly mineralized solutions with a high water absorption from 300 to 1000 g/g. In the base hydrophilic material, Aquapastus-11 (further abbreviated A11), an acrylic polymer matrix is filled by 23% (wt) biocatalytic wastes from the production of polyacrylamide. In the second innovative material, Aquapastus-22-Ag (A22-Ag or «black» gel), a similar polymer matrix was filled by 23% (wt) dispersed peat and by 1% silver ions, for the material’s resistance to biodegradation and osmotic stress. This hydrogel forms gel structures of the reinforced type which are more stable to pressure due to the fine-dispersed peat filler [41]. Descriptions of the synthesis, composition, and results of preliminary laboratory testing of the Aquapastus composites for soil conditioning are presented in our previous articles [23,41] and patents (see “Section 6”).

### 2.2. Hygroscopy, Water Retention, and Water Vapor Sorption Isotherm Analysis

Since the content of hygroscopic water in a material depends on the thermodynamic potential (activity) of water, it was necessary to obtain thermodynamic water retention curves (WRCs) for the tested materials. For this purpose, we used a combination of the traditional adsorption-static method and the new method of thermal desorption of water vapor, according to [41]. In the traditional adsorption-static method, samples of small mass (near 5 g), after preliminary spraying of a water aerosol, were exposed in an atmosphere saturated with water vapor in a desiccator with distilled water. After the adsorption equilibrium was established, they were transferred to desiccators with saturated solutions of K_2_SO_4_, KCl, and Ca(NO_3_)·4H_2_O salts, maintaining air relative humidity (water activity) of 0.98, 0.86, and 0.55, respectively. These values of water activity corresponded to the absolute values of the thermodynamic potential of water (|Ψ|) at room (293 K) temperature of 2734, 20,411, and 80,907 J/kg. Weighing the samples at each stage of the equilibrium desorption of water vapor made it possible to determine the hygroscopic water content (*W*_h_) and obtain the WRC points as a dependence of |Ψ|(*W*_h_). Further removal of hygroscopic water was carried out using the AND MX-50 humidity analyzer (A&D, Tokyo, Japan) by gradual heating at temperature stages of 30, 40, 50, 60, 70, 80, and 105 °C. This simple procedure allowed us to estimate the WRC in the region of low water activity (less than 0.5), including areas with an activity of 0.001–0.01 (relative air humidity 0.1–1%) that had not previously been studied using conventional methods such as the static sorption equilibrium or the dynamic dewpoint isotherm modes, implemented in modern automatic sorbtometers [42]. An alternative analysis, allowing the simultaneous study of many samples, was based on the use of a BINDER ED023-230V drying oven (BINDER GmbH, Tuttlingen, Germany), maintaining an accurate (±0.1 °C) temperature at a given level in the required range from 30 to 105 °C. In this case, samples of polymer materials were exposed in a drying oven at a given temperature until thermodynamic equilibrium (at least 2–3 h), and then were quickly (to avoid the reverse adsorption of vapor from laboratory air) weighed on an accurate (0.0001 g) laboratory scales OHAUS Pioneer PX224/E (OHAUS, Greifensee, Switzerland). The calculation of the thermodynamic potential based on the drying temperature data was carried out using the formula [43] obtained from the fundamental Clausius–Clapeyron equation for conditions of constant temperature and relative air humidity in an external thermodynamic reservoir or laboratory room (see “Section 3.1”). The calculation of hygroscopic water content was carried out according to the formula:

(1) $$W_{h},\left[\%\right]=100\frac{m_{t}-m_{s}}{m_{s}-m_{0}}$$

where *m_t_* (g) is the mass of a vial with hygroscopic material at a given drying temperature, *m_s_* (g) is a similar value at a temperature of 105 °C for the conditionally complete dehydration of the material, and *m*_0_, (g) is the mass of an empty vial. All experiments were performed at least in triplicate.

Experimental data for polymer superabsorbents have been published earlier, including the article [23] with a similar research topic. However, here we combined them with new data for biopolymers and used them together to substantiate a new methodology for estimating the specific surface area of polymers, which is completely different from the previous publication [23].

### 2.3. Additional Methods and Data Processing

To additionally control the dynamics of temperature and relative humidity (water activity) in the drying oven, programmable data loggers DS1923 (Dallas Semiconductor (Maxim), Dallas, TX, USA) were used. The content of organic carbon (*C*, [%]) in the samples was determined by means of coulometric titration using the AN-7529 analyzer (Gomel Plant of Measuring Devices, Gomel, Russia). The pH was analyzed using the combined EC/TDS/pH meter HANNA HI 98129 Combo (HANNA Instruments Deutschland GmbH, Fleringen, Germany). The approximation of experimental data was carried out in the S-Plot 11.0 program (Systat Software GmbH, Erkrath, Germany) using the Regression Wizard nonlinear regression package. Statistical processing was carried out in MS Excel 16 spreadsheets (Microsoft, Redmond, DC, USA) and in the R (3.5.3) program (RStudio PBC, Boston, MA, USA).

## 3. Results

### 3.1. Theoretical Basis for the Thermodynamic Estimation of SSA through the Hygroscopy of Materials

The theoretical dependence of the specific surface area and the hygroscopy of materials is based on three fundamental thermodynamic equations. The first is the well-known Gibbs formula relating the modulus (absolute values) of the thermodynamic potential of water (|Ψ|, J/kg) and relative air humidity (water activity, *f*) at constant temperature (*T*, K):

(2) $$\;\left|\Psi \right|=-\frac{RT}{M}\ln\left(f\right),$$

where *R* = 8.314 J/(mol·K) is a universal gas constant, and *M* = 0.018 kg/mol is the molar mass of water.

The second is the fundamental Deryagin equation for the disjoining pressure of water films of thickness (*h*, m), presented in the form of the equivalent thermodynamic potential of water, according to [41]:

(3) $$\left|\Psi \right|=a\cdot \exp\left(-\frac{h}{\lambda }\right)=a\cdot \exp\left(-bW\right),\;b=\frac{1}{S\rho_{l}\lambda },$$

where *a* (J/kg) is the physically based parameter reflecting the surface shape and potential (charge), λ (m) is the length of correlation for the structural forces or effective Debye thickness of the double electric layer for ion-electrostatic forces, *S* (m^2^/kg) is the variable specific surface of the interphase boundary, *b* (kg/kg) is the physically based parameter controlled by λ and *S*, *W* (kg/kg) is the water content of the material, and $\rho_{l}$ = 1000 kg/m^3^ is the density of water.

The third equation relates the thermodynamic potential of water to the temperature of the local heating (drying) of the sample (*T*_d_, K) in a large thermodynamic reservoir (laboratory room) with a constant temperature (*T*_r_, K) and relative humidity (*f*_r_), as a partial solution of the fundamental Clausius–Clapeyron equation obtained in [43]:

|Ψ| *= Q* − β·*T*_d_ (4)

where β *=* {*Q*/*T*_r_ − *R*·ln(*f*_r_)/*M*}, and *Q =* 2401 ± 3 kJ/kg is the specific heat of evaporation for the temperature range of 0–100 °C. Its verification using DS1923 air temperature and humidity loggers was given in [23].

Equation (4) allows us to estimate the extremely important indicator of the standard thermodynamic potential of water (Ψ_st_, J/kg) for conditionally “zero” water content (*W*) in the material after standard drying at 378 K (105 °C):

(5) $$\left|\Psi_{\mathrm{st}}\right|=Q-378\cdot \left(\frac{Q}{T_{r}}-\frac{R}{M}\ln(f_{r})\right)$$

**Figure 1:**
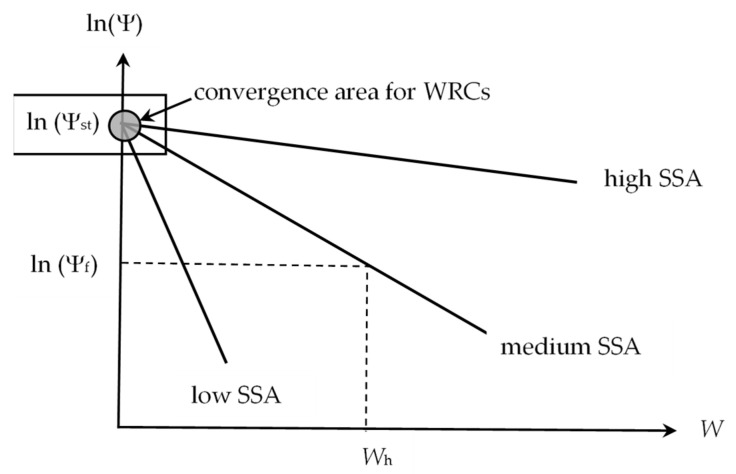
Thermodynamic water retention curves for materials with different SSA (tendency of convergence to Ψ_st_).

The value of Ψ_st_ determines the region of convergence of all thermodynamic water retention curves for materials at their conditionally zero moisture content (Figure 1).

According to the disjoining pressure model (3), on a semi-logarithmic scale, the water retention curves are transformed into straight lines converging at the point *W* = 0; Ψ = Ψ_st_. The greater the slope of the straight lines (parameter *b* in Equation (3)), the smaller the specific surface area of the material (SSA). From the stability conditions of thin films, it is easy to obtain the following fundamental expression for calculating SSA [41]:

(6) $$S=\frac{\sqrt{2\exp\left(-2\right)}}{br_{0}\rho_{l}},$$

where *r*_0_ = 1.38 × 10^−10^ m is the crystallochemical radius of the water molecule.

For a known hygroscopic water content (*W* = *W*_h_), the following geometric relationship is evident from Figure 1:

(7) $$b=\frac{\ln(\Psi_{\mathrm{st}})-\ln(\Psi_{f})}{W_{h}}=\frac{\ln\left(\Psi_{\mathrm{st}}/\Psi_{f}\right)}{W_{h}}$$

Substituting Expression (7) into Equation (6) gives the basic formula for the fundamental relationship between SSA and the hygroscopy of materials:

(8) $$S=\frac{\sqrt{2\exp\left(-2\right)}}{\ln\left(\Psi_{\mathrm{st}}/\Psi_{f}\right)\;r_{0}\rho_{l}}W_{h}$$

To quantify the ratio Ψ_st_/Ψ_f_, it is convenient to use the following combination of thermodynamic Equations (2) and (5):

(9) $$\frac{\Psi_{\mathrm{st}}}{\Psi_{f}}=\frac{T_{d}}{T_{r}}-\frac{MQ}{RT_{r}\ln(f)}\left(\frac{T_{d}}{T_{r}}-1\right)$$

For the temperature of the standard drying of materials (*T*_d_ = 398 K) and normal room temperature (*T*_r_ = 293 K), Expression (9) can be simplified taking into account the numerical values of the physical constants included in it:

(10) $$\frac{\Psi_{\mathrm{st}}}{\Psi_{f}}\approx 1.29-\frac{5.14}{\ln(f_{r})}$$

Then, the simplified formula for estimating SSA will look like this:

(11) $$S=\frac{\sqrt{2\exp\left(-2\right)}}{\ln\left(1.29-\frac{5.14}{\ln\left(f_{r}\right)}\right)r_{0}\rho_{l}}W_{h}\approx \frac{37.7}{\ln\left(1.29-\frac{5.14}{\ln\left(f_{r}\right)}\right)\;}W_{h},$$

where 37.7 is a parameter that takes into account all the numerical constants included in Equation (11). Here, *S* (m^2^/g); *W*_h_,(%); and *f* is dimensionless (relative humidity).

Formula (11) for estimating SSA, as can be seen, uses only two predictors—the content of hygroscopic water in the material (*W*_h_) and the equilibrium relative humidity of the air (water activity, *f*_r_) in the room where the materials under study are located. If data on water activity are not available, then, in a rough approximation for a humid climate, the following linear relationships can be used to estimate SAA, assuming that relative air humidity is 30% in winter (more precisely, the cold period with the heating of the room) and 50% in summer (the warm period without the heating of the room):

(12) $$S\approx 21.98\cdot W_{h}\mathrm{(winter)}$$

(13) $$S\approx 17.42\cdot W_{h}\mathrm{(summer)}$$

**Figure 2:**
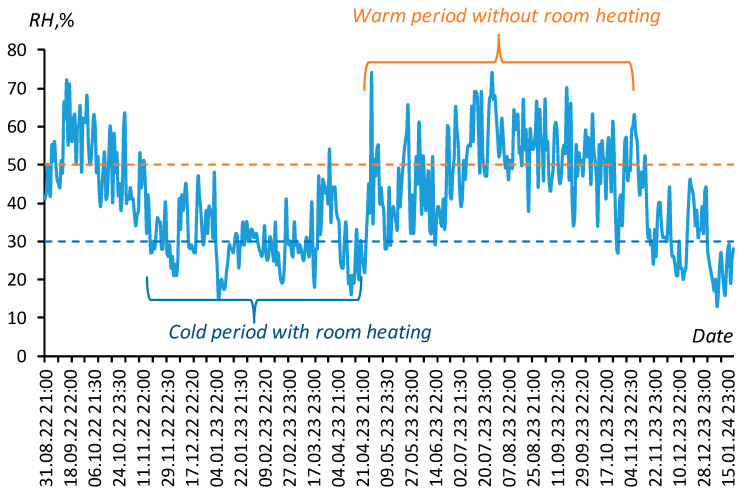
Daily monitoring of relative air humidity in the ILAN Laboratory (Moscow, Russia, 2022–2024). Blue and orange dotted lines mark air humidity 30% and 50%.

Figure 2 summarizes the results of monitoring the dynamics of relative air humidity (*RH*, [%] = 100·*f*_r_) in the ILAN Laboratory (Moscow, Russia), confirming this choice. The characteristic values of relative humidity of 30% and 50% in the cold period with room heating and in the warm period without it are shown in the figure with a dotted line.

In general, the specific surface of the material in m^2^/g is approximately 20 of its hygroscopic water content in %.

The new approach was compared with the standard BET method, using a physically based BET model to estimate the water content of the adsorption monolayer (*W*_m_):

(14) $$W=\frac{nW_{m}f}{\left(1-f\right)\cdot \left(1+\left(n-1\right)f\right)},$$

where *n* is a dimensionless approximation parameter, and *f* is water activity. The calculation of SSA by the *W*_m_ value is based on the well-known equation [1]:

(15) $$S_{\mathrm{BET}}=\frac{W_{m}N_{A}s_{0}}{M}\approx 3515\cdot W_{m}$$

where *N*_A_ = 6.02⋅10^23^ mol^−1^ is the Avogadro number, and *s*_0_ (m^2^) is the cross-section area of the water molecule, which can be determined from the following equation [1]:

(16) $$s_{0}=1.091\cdot \sqrt[{3}]{{\left(\frac{M}{\rho \cdot N_{A}}\right)}^{2}}$$

where 1.091 is the packing factor for the 12 nearest molecules of the liquid phase and 6 molecules on a flat surface for the solid phase of the adsorbent. Equation (16) gives for a water molecule s_0_ = 0.105 nm^2^ and, accordingly, the constant 3515 in Formula (15).

It is important to note that hygroscopic assessment, like any sorption method, including standard BET analysis, is determined by the total surface energy of the adsorbent. This means that not only the estimated specific surface area of the material, but also its quality (charge, the density of adsorption centers, and their affinity for the adsorbate) will affect the results of such an assessment. Therefore, in comparative studies, it is better to use materials with similar surface quality characteristics. That is, hydrophilic polymers can be compared with each other, but not with hydrophobic materials, where low hygroscopicity does not guarantee a small specific surface area. Also, a prerequisite for the new method is the achieved balance of water content in the material and the relative humidity of the surrounding air, characterized by the hygroscopicity of the polymer. Wet or overdried materials cannot be studied until their water content reaches this balance.

### 3.2. Experimental Results; Mathematical and Statistical Processing

**Figure 3:**
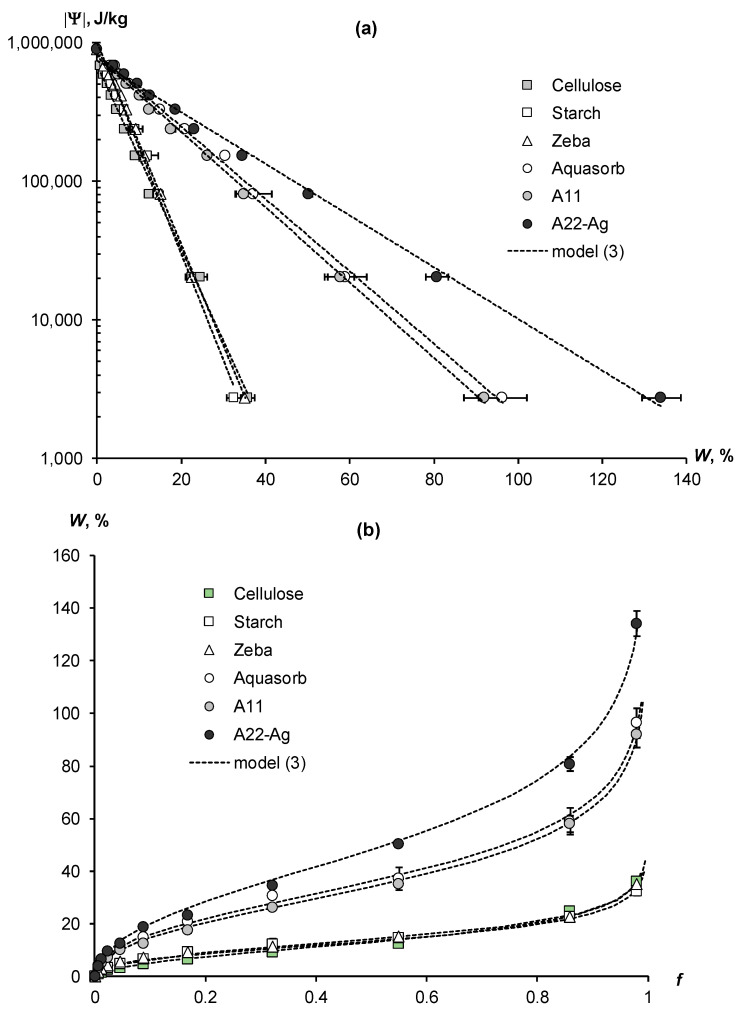
Experimental thermodynamic WRCs (**a**) and water vapor desorption isotherms (**b**) of natural biopolymers and synthetic polymer composites and their approximation by the disjoining pressure model (3). Here and further, the error bars are confidence intervals at *p*-value = 0.05.

Figure 3a presents the experimental WRCs of biopolymers and synthetic composite superabsorbents with an acrylic polymer matrix. The results of their transformation into water vapor desorption isotherms using the fundamental thermodynamic Gibbs formula (equation inverse to (2)) are shown in Figure 3b. In both figures, the experimental data are fitted by the disjoining pressure model (Equation (3)) in the form of dotted lines. The arrangement of experimental data and model lines in Figure 3a are in full accordance with the thermodynamic theory of water retention, which predicts the convergence of WRCs in the region of the standard thermodynamic potential of water (Ψ_st_) at conditionally “zero” water content (Equation (5); schema in Figure 1). The innovative superabsorbent A22-Ag, based on an acrylic matrix filled with dispersed peat and silver ions, demonstrated the greatest hygroscopicity. Its water retention curve sharply differed from the WRCs of other polymers and was characterized by a minimal slope to the abscissa axis (Figure 3a). The content of hygroscopic water at water activity *f* = 0.98 or the so-called “maximum hygroscopicity” (MH) in this polymer composite reached 134 ± 5%, i.e., exceeded more than 1.3 times the mass of the adsorbent itself in a dry state.

Two other superabsorbents, Aquasorb and A11, had similar water retention characteristics in the form of WRCs and water vapor desorption isotherms located in the middle part of Figure 3a,b. The WRCs slopes of these materials were steeper than those of the A22-Ag composite, and their maximum hygroscopy, respectively, was less. The Aquasorb brand has MH = 96 ± 5%, and the A11 material has MH = 92 ± 5%. The remaining three materials—biopolymers (cellulose and starch) and the Zeba composite—were grouped by water retention into one cluster, occupying the lower position in Figure 3a,b. Their WRCs had maximum slopes, and MH indicators, respectively, had minimum values from 32 ± 2% (starch) to 35 ± 2% (the Zeba composite) and up to 36 ± 2% (cellulose).

For all polymer materials, the experimental water retention data were adequately fitted by the disjoining pressure model (Equation (3)). The approximation parameters for WRCs and their statistical assessment are given in Table 2. High coefficients of determination (R^2^ = 0.995–0.999) and low standard errors of estimation (s = 8–22 kJ/kg) confirm the good correspondence of experimental data to theoretical Equation (3). The parameter *a* associated with the standard potential of conditionally zero water content (Ψ_st_) varied from 743 to 999 kJ/kg. The slope WRC index (*b*) was minimal for the composite superabsorbent A22-Ag (4.3 ± 0.3 g/g) and maximal for the cellulose biopolymer (18.2 ± 0.4 g/g). Since the SSA values are the inverse of the WRC slope index (see Equation (6)), the microcrystalline cellulose biopolymer was characterized by a minimum specific surface area (207 ± 5 m^2^/g), and the A22-Ag superabsorbent had a maximum SSA (879 ± 24 m^2^/g). Other polymers occupied an intermediate position in terms of the compared WRC slope and SSA indicators. Both compared indicators for the starch biopolymer and the Zeba composite material with an acrylic polymer matrix filled with starch were similar (Table 2). Also, the *b* parameters and the SSA indicators calculated by them did not differ statistically significantly for both composite materials—the Aquasorb and the A11 superabsorbent.

**Figure 4:**
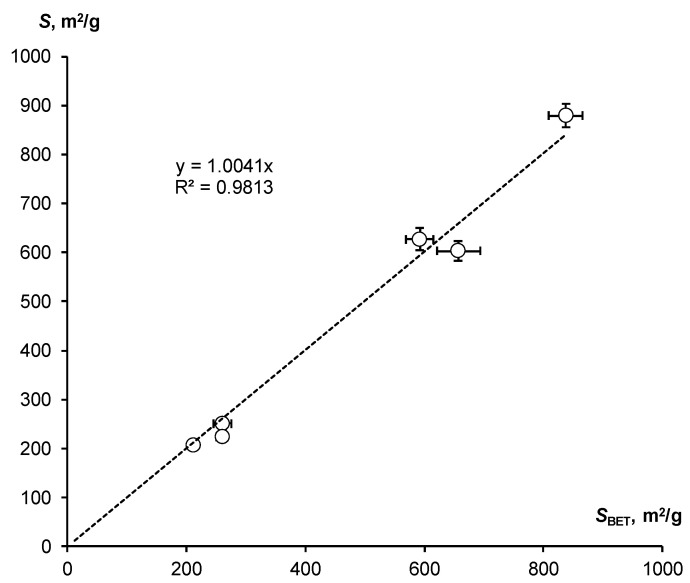
Correlation of SSA estimates by means of the WRC slope and the standard BET method.

The assessment of SSA by means of the WRC slope was compared with the standard BET method (*S*_BET_). Both estimates agree well with each other within the normal range of variations (Table 2). Their comparison is shown in Figure 4.

Despite the scatter of data, the entire series under study is located closely along a straight line with a slope equal to 1, which confirms the close correlation (R = 0.991) of the compared indicators and their actual equality.

**Figure 5:**
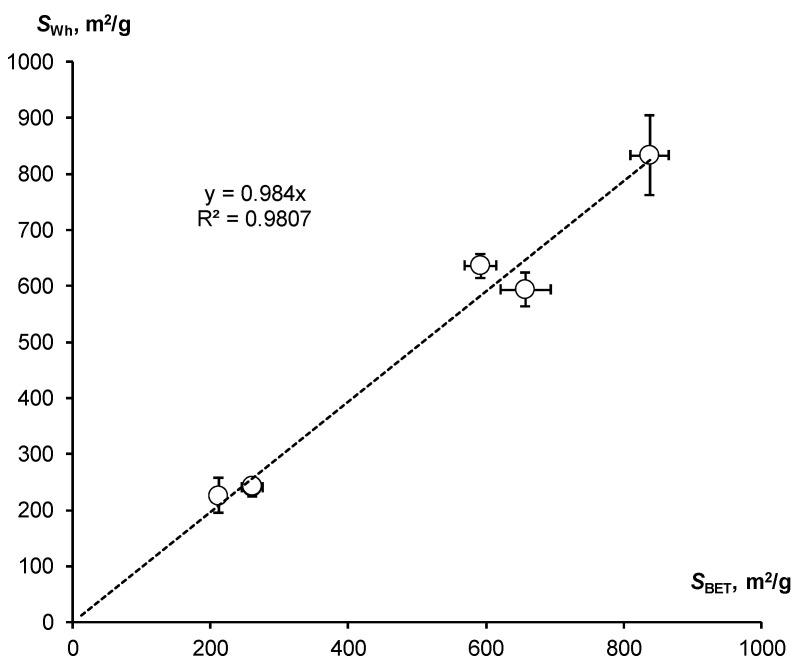
Correlation of SSA estimates by means of hygroscopic water content in the water activity range from 0.33 to 0.98, and the standard BET method.

Using theoretical Equation (11) and experimental data from water vapor desorption isotherms (Figure 3b) in the water activity range from 0.33 to 0.98, we estimated SSA using a new method based on the hygroscopic water content (the *S*_Wh_ index). The averaged estimates with confidence intervals (*p*-value = 0.05) are compared with the results of the standard BET method in Figure 5.

It also shows a close correlation of the compared indicators with the angular coefficient of the linear regression line (0.984) close to unity and the high Pearson correlation coefficient (R = 0.990). Consequently, the estimation of the specific surface area by means of hygroscopy based on a new methodological approach also actually coincides with the standard BET estimation for all studied polymers.

**Figure 6:**
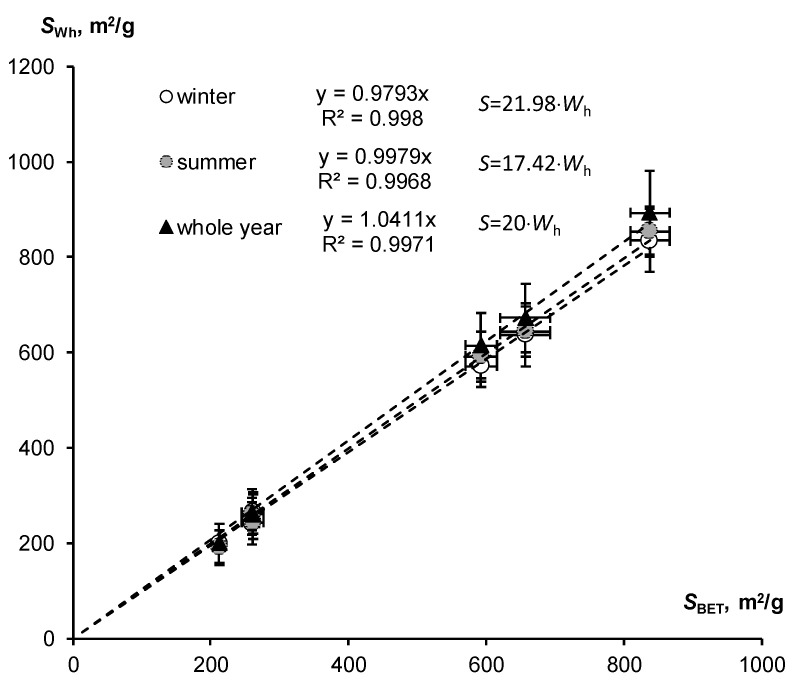
Comparison of SSA estimates by means of hygroscopic water content in winter, summer and whole year with the standard BET method (SSA calculation using simplified Formulas (12) and (13) and the approximate rule: *S* ≈ 20·*W*_h_).

Similar results were obtained for SSA estimates based on data on hygroscopic water content for a fixed water activity (air humidity) in the winter and summer seasons, using Equations (12) and (13), as well as the approximate rule: *S* ≈ 20·*W*_h_. The comparison of these estimates with the standard BET method is shown in Figure 6. All regression lines were characterized by slope coefficients close to unity (from 0.979 to 1.04), with close correlation reflected by Pearson coefficients from 0.998 to 0.999. Thus, simplified SSA assessment methods using fixed air humidity of 30% and 50% for the winter and summer seasons, as well as the orienting rule “twenty hygroscopic water content”, were also quite comparable with the results of the standard BET method.

The standard analysis of variance (ANOVA) results also confirmed the absence of statistically significant differences between the new approach and the standard BET method for determining the specific surface area. Since the preliminary test for the normality of the distribution of SSA indicators in the form of the Pearson, Lilliefors (Kolmogorov–Smirnov), and Shapiro–Wilk criteria was not passed (*p*-values from 9.2 × 10^−7^ and less), and the Bartlett test of homogeneity of variations for the factor “SSA evaluation methods”, on the contrary, was passed at *p*-value = 0.951, we decided to use both parametric and non-parametric ANOVA. The parametric analysis of variance (*p*-value = 0.984) and non-parametric Kruskal–Wallis rank-sum test (*p*-value = 0.738) both showed the absence of a statistically significant effect of the compared methods on the SSA assessment. A more detailed multiple comparison of Tukey averages (parametric) and pairwise comparisons using the Wilcoxon rank-sum test are given in Table 3. Their analysis confirms the absence of statistically significant pairwise differences between the compared variants of the new methodological approach and the standard BET method. In general, a new approach with a simplified assessment of the hydrophilic surface of polymers by means of their hygroscopy can be successfully used instead of the more time-consuming and expensive standard BET method without the risk of a statistically significant distortion of the estimate at the accepted level of *p*-value = 0.05.

## 4. Discussion

### 4.1. Comparison of the Obtained Results with Known Data

#### 4.1.1. Hygroscopicity of Biopolymers and Synthetic Polymers

The results correspond to the known data on the quantification of water vapor desorption isotherms and hygroscopy for the studied biopolymers and composite superabsorbents based on an acrylic polymer matrix. In particular, the water vapor sorption isotherms of cellulose fibers from [21] had a hygroscopic water content from 3–5% to 20–30% in the water activity range of 0.1–0.9, which is close to our data (Figure 3b). Similar characteristics of water sorption for starch isolated from cassava (*Manihot esculenta* Crantz) are given in [22]. The maximum values *W*_h_ for starch, as for cellulose, did not exceed 30–33% with water activity close to 1. Similar values of water vapor sorption capacity for different polysaccharides are given in [44]. For potato starch, maximum *W*_h_ values varied from 40 to 45%, and for microcrystalline cellulose, wood pulp, and cotton fibers, the *W*_h_ range was from 18 to 32%. Other polysaccharides from [44] had *W*_h_ ranges of 15–25% (chitin and chitosan), 30–36% (pectin), and 32–50% (xylan).

Synthetic polymer superabsorbents and biopolymer composite hydrogels have higher hygroscopicity compared to pure biopolymers [13,23,45,46,47]. For example, the hygroscopicity of superabsorbents with an acrylic polymer matrix can exceed 80–100% [41,45,48,49], which is in good agreement with our results (Figure 3b). Polysaccharide hydrogels with a biopolymer matrix based on starch, chitosan, or alginate, as well as metal- and halide-free, polymeric water vapor sorbents with embedded acetate, oxalate, and citrate anions also demonstrate a quite high water sorption capacity of 40–60% or more [41,48,49]. However, the most effective method of increasing the hygroscopicity of polymer composites is the incorporation of hydrophilic metal cations, such as Li^+^, Ca^2+^, Na^+^, and Ag^+^, into the polymer matrix, or the placement of highly hygroscopic salts, mainly chlorides (LiCl, CaCl_2_, MgCl_2_, polypyrrole chloride, etc.) in the polymer network [13,41,45,46,48,50,51]. In the case of embedding hydrophilic cations, the water vapor sorption capacity of composite superabsorbents increases to 100–140% or more, regardless of the polymer matrix (synthetic polymers or natural biopolymers-polysaccharides) [41,48]. In our A22-Ag composite, the incorporation of silver ions increased the water vapor sorption capacity up to 30–40% at low water activity (water vapor sorption capacity 0.2–0.3) and up to 140% in a water-saturated atmosphere (*f* = 0.98) (Figure 3b).

An even greater water absorption effect occurs when highly hygroscopic salts are introduced into the polymer network of composite superabsorbents. Lithium chloride’s own water vapor sorption capacity exceeds 800% (8 g H_2_O per 1 g of dry salt) in a wet atmosphere (*f* = 0.85–0.90) [51]. Embedding a 25% (from saturation) solution of lithium chloride into the polymer network of a polyacrylamide hydrogel leads to water vapor sorption isotherms of the composite superabsorbent practically no different from those for pure lithium chloride [51]. The maximum hygroscopy of the PAA-Li superabsorbent reached 800%, and water vapor sorption capacity in a dry atmosphere (*f* = 0.2–0.3) was around 150–200% [51]. A similar lithium chloride superabsorbent, with a hybrid biopolymer matrix based on konjac glucomannan, and hydroxypropyl cellulose had a slightly lower, but also extremely high water vapor sorption capacity, reaching 60–100% in a dry atmosphere (*f* = 0.15–0.3) and more than 200–250% in the humid air with water activity *f* = 0.6 and higher [13]. Calcium chloride polymer superabsorbents reduce water vapor sorption capacity compared to lithium chloride hydrogels, but it still remains very high and can reach 40–110% in the humid air (*f* = 0.6) and 30–70% in the relatively dry air (*f* = 0.35%), using the example of an acrylic composite PAM-CNT-CaCl_2_ from [45]. This decrease is quite understandable from a comparison of the hygroscopic points (water activity in a saturated solution) for lithium chloride (*f* = 0.11 ± 0.03) and calcium chloride (*f* = 0.29 ± 0.03) under normal laboratory temperature conditions [52]. In general, the technology for producing super-hygroscopic polymer composites using the swelling of synthetic hydrogels, less often biopolymer gels in aqueous solutions of lithium or calcium chlorides, is, apparently, the most effective in the case of the absorption of water vapor from the air. However, such composites sharply lose their ability to swell in pure aqueous solutions and can no longer be superabsorbents of liquid water necessary for soil conditioning [41]. The combination of high hygroscopicity and liquid water absorption (swelling in pure water and weakly mineralized solutions) can be achieved by introducing monovalent metal cations (lithium, sodium, silver, etc.) into the polymer matrix, as, for example, in the innovative material A22-Ag (Table 1, Figure 3b).

#### 4.1.2. Specific Surface Area of Biopolymers and Synthetic Polymers

The known estimates of the specific surface area of biopolymers and synthetic polymer composites are quite contradictory and strongly depend on the method of determining the SSA index and further data processing. In [22], estimates of the monolayer for different types of starch using water vapor sorption isotherms and their treatment with the BET and the GAB models varied in the range of 4.7 ± 2.0–9.4 ± 3.8 g/g for the standard BET method and from 8.7 ± 0.2 g/g to 11.2 ± 0.3 g/g in the case of data processing using the GAB model. Using Formula (15), it is easy to see that for the standard BET method, this variation of the monolayer gives a range of SSA from 165 ± 70 m^2^/g to 330 ± 133 m^2^/g, which is in principle close to our estimates for pure starch and starch-containing Zeba composite from 225 ± 7 m^2^/g to 261 ± 15 m^2^/g (Table 2). However, the use of the GAB model, reducing the variation (standard error of estimation), greatly increases the SSA value of starch up to 300–400 m^2^/g according to [22]. As in our study (Equation (15)), [22] used a physically based approach [1], estimating the cross-section area of a water molecule at 0.105 nm^2^. Note that the current standard [18] suggests using a higher value of *s*_0_ = 0.125 nm^2^ for a water molecule. It is clear that such a discrepancy in *s*_0_ estimates may be the reason for a significant variation (1.2 times) in the SSA estimates for studies using different approaches. Therefore, it is very important to specify not only the physical model for calculating monolayers (Langmuir, BET, GAB, etc.), but also the cross-section area of the adsorbate molecule.

Even higher differences in the SSA estimates arise when using the standard BET method based on the low-temperature adsorption of nitrogen. As mentioned in the Introduction, the standard BET method using nitrogen gives a specific surface area for starch of about 0.3 m^2^/g [20], or almost 1000 times less than in the case of using water molecules [22]. Slightly higher standard estimates of SSA (11–22 m^2^/g) using nitrogen sorption are given in [8] for chitosan and metal–organic composites based on it. And only surface-modified biopolymers, such as cellulose aerogels with a fibrillar structure, have a sufficiently high specific surface area (up to 75–143 m^2^/g), determined by means of the standard BET method with nitrogen sorption [21]. A comprehensive study [44] compares SSA estimates with water and n-hexane sorption. Cellulose and cellulosic material after chemical treatment (microcrystalline cellulose, cotton, mercerized fibers, Kraft sheets, and viscose fibers) had a specific surface area of 84–200 m^2^/g (water) and 3–7 m^2^/g (n-hexane). For starch, SSA was 231 m^2^/g (water) and 4.6 m^2^/g (n-hexane). Other polysaccharides (chitin, chitosan, pectin, xylan, and mannan) had similar SSA characteristics in the range of 90–242 m^2^/g (water) and 5.2–6.8 m^2^/g (n-hexane). In general, that study [44] fully confirms the large (10–60 times and higher) differences in the assessment of SSA by means the standard BET method when using different adsorbates with polar and non-polar molecules.

Synthetic polymer composites with an acrylic matrix have a higher SSA both in the case of H_2_O sorption and when using the standard BET method with low-temperature nitrogen sorption. Lee et al. [2] reports a specific surface area of 70–130 m^2^/g determined by nitrogen sorption for silicon–polymer composites with butyl-methacrylate copolymer. Higher estimates using nitrogen sorption were obtained in [53] for acrylic acid (SSA = 360–390 m^2^/g) and acrylamide (370–382 m^2^/g). However, they are 1.6–2.4 times lower than the SSA values estimated from water sorption for the studied polymer superabsorbents with an acrylic matrix, excluding the Zeba composite (Table 2).

### 4.2. Estimation of Specific Surface Area Using Hygroscopy

Only a few publications analyze the relationship between hygroscopy and the specific surface area of polydisperse materials. In polymer chemistry, we found one article [3] showing a close (R^2^ = 0.934) linear regression between the hygroscopicity of cotton fibers containing 90–99% cellulose and their specific surface area, determined by the sorption of methylene blue in liquid phase. In this study, the hygroscopic water content range of 5.8–9% corresponded to an SSA range of 28 to 53 m^2^/g. A larger number of publications on this topic can be found in the geosciences, in particular, in soil science. The article [28] analyzes the water retention curves (transformed water vapor sorption isotherms) of Danish soils with a wide range of clay and organic carbon contents, measured by a chilled-mirror dew point psychrometer. The slope of the water retention curves in semi-logarithmic coordinates (*b*) and the specific surface area (direct estimation by means of the ethylene glycol monoethyl ether method and calculation based on the thickness of the film of adsorbed water) were in the following inverse relationship: *b* = SSA^−*n*^. For SSA, estimated from the thickness of the water film, the *n* value was close to 1 (*n* = 0.96). This important empirical result [28] fully corresponds to our theoretical concept linking the slope of the water retention curves and the specific surface area through fundamental Equation (6).

The study [30] confirmed that a single measure of hygroscopic water content can provide reliable estimates of the SSA. It proposed regression models to estimate the SSA index from *W*_h_ for three soil sample groups with different clay and soil organic matter contents. Previously, Wuddivira et al. [54] showed that *W*_h_ can be a good predictor of these SSA-controlling factors, in particular, clay content. The linear function of the water vapor sorption isotherm (water retention curve) is defined by its slope and a fixed endpoint at zero water content (Figure 3a). The reciprocal of the slope as shown in [29] has a strong correlation with the clay fraction. However, the slope of the water retention curve has a fundamental relationship with the SSA parameter, according to Equation (6). Consequently, a strong correlation can be expected between *W*_h_ and specific surface area.

Indeed, the analysis of more than 300 soil samples in [30] revealed a close (R^2^ = 0.7–0.95) linear relationship between *W*_h_ and SSA in the form of SSA = *k·W*_h_ + *m*, where *k* and *m* are empirical parameters. The parameter *m* in some cases was close to zero or took negative values. Its average value for the range of water activity from 0.05 to 0.9 was *m* = 9.9 ± 16.8, i.e., it was not statistically significantly different from zero. The parameter *k* varied in a wide range from 7.5 to 97.5 m^2^/(g·%), with an average value of *k* = 32.2 ± 17.8. These results obviously do not contradict our theoretical approach (Formulas (11)–(13)). The coefficient *k*, theoretically predicted from Equation (11) for the range of water activity variation from 0.05 to 0.9 (from [30]), gives *k* = 19.1 ± 6.9, which fits well into the confidence interval *k* = 32.2 ± 17.8 in [30]. The strong (32.2/19.1 = 1.7 times) difference in the average values can, in our opinion, be caused both by the use of linear regression with the intercept in [30] and by the assessment of SSA through the sorption of dye rather than water vapor.

Also, [30] takes into account the hysteresis of the water vapor of sorption isotherms; therefore, the parameter *k* has different values for the adsorption and desorption of water vapor. On average, *k* = 29.5 ± 16.4 m^2^/(g·%) for desorption was 1.2 less than for adsorption. From our point of view, it is incorrect to consider the surface of dispersed particles unchanged in cycles of the wetting/drying of the material [41]. If, during the process of drying (the desorption of water vapor), the water content of a polydisperse material decreases to the limit of aggregative stability of colloids, and the total coagulation of colloidal particles causes a decrease in the specific surface area. The reverse process of water vapor adsorption does not restore the original dispersion (initial SSA), since this requires interaction with liquid water with a corresponding disjoining pressure that destroys the coagulation contacts of the particles. This is one of the physical mechanisms of sorption hysteresis and the reason for the differences in the specific surface area determined from the desorption branch (large SSA) compared to the adsorption branch (small SSA). Since the analysis of water vapor desorption gives higher (true) values of the specific surface area, we used temperature desorption to obtain thermodynamic water retention curves and estimate the SSA from them. However, the content of hygroscopic water may depend on the history of interaction of the material with the atmosphere (the adsorption or desorption of water vapor). Therefore, the question of the influence of sorption hysteresis on the assessment of SSA from the hygroscopy of materials remains open.

### 4.3. Independent Validation of a New Approach for Assessing SSA Using Hygroscopy

**Figure 7:**
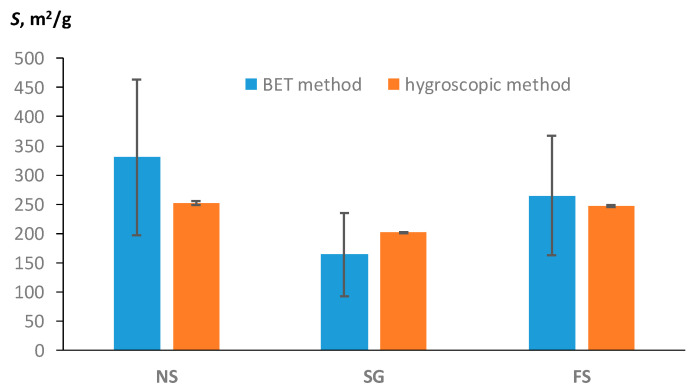
Comparison of SSA estimates by means of hygroscopic water content with the standard BET method (experimental data from [22]).

Among the available publications, only [22] contained the necessary set of data on the hygroscopic water content, water activity, and specific surface area of polymers, estimated using the BET method. Three types of starch—native (NS), granulate (SG), and fermented (FS)—were studied in [22]. The monolayer water content of these polymers according to the BET model was 9.3989 ± 3.7904 g/g (NS); 4.6637 ± 2.0328 g/g (SG); and 7.5257 ± 2.9108 g/g (FS). The hygroscopic water content and water activity for these types of starch were *W*_h_ = 14.347 ± 0.552 g/g at *f* = 0.492 (NS); *W*_h_ = 10.108 ± 0.081 g/g at *f* = 0.38 (SG); and *W*_h_ = 13.524 ± 0.232 g/g at *f* = 0.457. Using Equation (15), we calculated the specific surface area from the data on the monolayer of water in accordance with the BET model. A new approach based on theoretical Formula (11) allowed us to obtain an alternative estimate of SSA using data on the content of hygroscopic water and air humidity (water activity). Both estimates are compared in Figure 7.

The water monolayer, and therefore the SSA estimate from the BET model, had a larger standard error (vertical bars in the diagrams) than the SSA estimate from the new method using hygroscopic water content. Hence, despite the discrepancy between the average values, both methods give a close estimate of SSA, taking into account the variation in the data. This conclusion about the absence of statistically significant differences between the two estimates of SSA is confirmed by the standard *t*-test for dependent samples used for the pairwise comparison of the two methods for each type of starch. The preliminary Shapiro–Wilk *W*-test confirmed the normality of the distribution of pairwise differences for the studied samples, and, accordingly, the use of a parametric comparison of averages. The *p*-values were 0.165, 0.172, and 0.164 for the NS, SG, and FS samples, respectively. A pairwise comparison of the averages using the *t*-test showed the absence of statistically significant differences at *p*-values = 0.238 (NS), 0.286 (SG), and 0.711 (FS), significantly greater than the reference *p*-value = 0.05. Since the average values of SE estimates for the new hygroscopic method and the standard BET method did not differ statistically significantly, both methods gave equivalent results for independent validation, as well as in our experimental verification (Figure 4, Figure 5 and Figure 6, Table 3).

In general, the new physically based approach provides an assessment of SSA that does not contradict the standard BET method, significantly simplifying the obtaining of information on the hydrophilic specific surface area of polymers, as a basic characteristic of their surface activity. The new method does not require expensive equipment like sorbtometers for BET analysis. It can be implemented in any laboratory equipped with a drying oven and scales. Although the time for assessing hygroscopicity (6–12 h of standard drying at 105 °C) is comparable to the time for obtaining sorption isotherms in BET analysis, the determination of hygroscopicity can be carried out simultaneously in many samples. As a result, the new hygroscopic method can be considered rapid compared to standard BET analysis, with a significant time gain for serial studies. In addition, the analysis of hygroscopicity is mandatory and precedes any study of the composition and properties of polymer materials. The new methodological approach opens up the possibility of using these usually routine data to estimate the specific surface area of materials.

## 5. Conclusions

In this study, we proposed that the determination of the hygroscopic water content of polymers at a fixed relative humidity (water activity), which is time- and cost-effective and necessary in any chemical analysis, can be used for the physically based assessment of specific surface area instead of the standard BET method with expensive sorbtometric equipment. The thermodynamic theory of water retention, including the fundamental dependences of the water potential on air humidity and temperature, and Deryagin’s theory of disjoining water pressure provided physically based formulas for calculating the specific surface area from the data of hygroscopic water content. This approach, apparently, was proposed for the first time, in contrast to the known empirical models linking hygroscopy and the specific surface area of materials. Its practical testing for natural polysaccharide polymers (monocrystalline cellulose and starch), as well as synthetic composite superabsorbents based on an acrylic polymer matrix, gave satisfactory results. A comparative statistical analysis of the results of the new approach and the standard assessment of the specific surface area by means of the BET method using water vapor sorption isotherms did not reveal significant differences between the compared methods at the level of the generally accepted 95% probability. The same result was obtained on independent research material for different types of starch. This research allows us to conclude that a new approach to the assessment of the hydrophilic specific surface area of polymers using data on their hygroscopy in an atmosphere with known air humidity gives quite acceptable results in comparison with the standard BET method, which fit into the range of permissible variation of the sorption equilibrium data. The new methodology compares favorably with the standard SSA assessment, using water vapor sorption isotherms, due to its temperature invariance. The influence of sorption hysteresis on the results of sorption assessment of the specific surface remains an open question, which may become one of the future challenges in this area.

## 6. Patents

The results of the work were used in the technology of filled hydrogels (superabsorbents) for soil conditioning, patented in the Russian Federation:

Patent RU 2726561 (https://findpatent.ru/patent/272/2726561.html, accessed on 22 May 2023);

Patent RU 2639789 (http://www.findpatent.ru/patent/263/2639789.html, accessed on 22 May 2022).

## Figures and Tables

**Table 1:** Composition and some properties of the tested materials.

Polymeric Materials	Composition	*C*%	*W*_h_^S^%	*W*_h_^W^%	*pH*
**Biopolymers (polysaccharides):**					
Starch	(C_6_H_10_O_5_)n 99%; ash < 0.4%	44.1 ± 0.4	14 ± 2	12 ± 2	5.8 ± 0.5
Cellulose microcrystalline	(C_6_H_10_O_5_)n 99%; ash < 0.05%	42.0 ± 0.5	11 ± 2	9 ± 2	6.5 ± 0.5
**Synthetic composites (acrylic-based superabsorbents):**					
Aquasorb	AcK, PAA *,	39.5 ± 0.5	37 ± 3	29 ± 3	7.3 ± 0.1
Zeba	PAA, AcK, starch	46.6 ± 0.7	15 ± 2	11 ± 2	7.0 ± 0.1
A11	Ac NH_4_, PAA, PAA-biocatalysis waste	45.0 ± 0.6	34 ± 3	26 ± 2	7.2 ± 0.1
A22-Ag	AcNa, PAA, peat, silver	47.5 ± 0.3	49 ± 3	38 ± 3	7.4 ± 0.1

* Denotation: PAA—polyacrylamide; AcK, AcNa, AcNH_4_—potassium, sodium, and ammonium acrylates; here and below, ± is the confidence interval at *p*-value = 0.05.

**Table 2:** Approximation parameters and their statistics for the disjoining pressure model and the standard BET model.

Materials	Model (3): |Ψ| = *a∙*exp(–*b∙W*)				Model BET: *W* = *n∙W*_m_*∙f*/{(1–*f*)*∙*(1 + (*n*–1)*∙f*}					
	R^2^	s, [kJ/kg]	*a*, [kJ/kg]	*b*, [g/g]	R^2^	s	*W*_m_, [%]	*n*	*S,* [m^2^/g]	*S*_BET_, [m^2^/g]
Cellulose	0.999	8.4	778.0 ± 8.8	18.2 ± 0.4	0.989	0.5	6.1 ± 0.2	26.2 ± 4.7	207 ± 5	213 ± 7
Starch	0.996	16.8	861.5 ± 22.2	15.0 ± 0.6	0.954	1.1	7.4 ± 0.4	39.1 ± 13.9	251 ± 11	261 ± 15
Zeba	0.998	15.4	998.5 ± 12.5	16.8 ± 0.4	0.979	0.7	7.4 ± 0.3	55.5 ± 13.0	225 ± 7	260 ± 10
Aquasorb	0.995	21.9	822.8 ± 16.7	6.0 ± 0.2	0.965	2.5	18.7 ± 1.0	30.4 ± 9.8	603 ± 20	657 ± 36
A11	0.996	19.7	789.7 ± 16.3	6.3 ± 0.2	0.982	1.6	16.8 ± 0.7	30.0 ± 6.8	627 ± 23	592 ± 23
A22-Ag	0.995	21.8	742.5 ± 16.8	4.3 ± 0.3	0.995	1.2	23.8 ± 0.5	24.8 ± 3.0	879 ± 24	897 ± 28

**Table 3:** Parametric and nonparametric ANOVA tests for the pairwise comparison of different SSA assessment methods.

Pairs of Compared Methods	Tukey’s Test, *p*-Values	Wilcoxon Test, *p*-Values
WRC–*W*_h_ *	0.998	0.790
BET–*W*_h_	0.983	0.794
BET–WRC	0.993	0.791

* Denotation: WRC–SSA assessment by means of the slope of the WRC; BET—the BET method; *W*_h_–SSA assessment by means of polymer hygroscopy.

## Data Availability

The data presented in this study are available on request from the corresponding author.

## References

[1] Gregg S.J., Sing K.S.W. (1982). Adsorption, Surface Area and Porosity.

[2] Lee V.A., Craig R.G., Filisko F.E., Zand R. (1996). Preparation and characterization of high-surface-area polymer substrates for microcalorimetry. J. Biomed. Mater. Res. A.

[3] Kaewprasit C., Abidi N., Gourlot J.P. Effect of Adsorbed Water on the Specific Surface Area of some Standards Cotton. Proceedings of the Beltwide Cotton Conference, National Cotton Council.

[4] Gu C., Liu D., Huang W., Liu J., Yang R. (2015). Synthesis of covalent triazine-based frameworks with high CO_2_ adsorption and selectivity. Polym. Chem..

[5] Dai J.F., Wang G.J., Ma L., Wu C.K. (2015). Surface Properties of Graphene: Relationship to Graphene-Polymer Composites. Rev. Adv. Mater. Sci..

[6] Mansurov Z.A. (2020). Carbon Nanomaterials in Biomedicine and the Environment.

[7] Jiang J.-X., Su F., Trewin A., Wood C.D., Niu H., Jones J.T.A., Khimyak Y.Z., Cooper A.I. (2008). Synthetic Control of the Pore Dimension and Surface Area in Conjugated Microporous Polymer and Copolymer Networks. J. Am. Chem. Soc..

[8] Iwuozor K.O., Ighalo J.O., Emenike E.C., Igwegbe C.A., Adeniyi A.G. (2021). Do adsorbent pore size and specific surface area affect the kinetics of methyl orange aqueous phase adsorption?. J. Chem. Lett..

[9] Gong L. (2021). A superhydrophobic and porous polymer adsorbent with large surface area. J. Mater. Chem. A.

[10] Rothon R. (2017). Fillers for Polymer Applications.

[11] Zhang L., Ma J., Lyu B., Zhang Y., Gao D., Liu C., Li X. (2020). Mitochondrial structure-inspired high specific surface area polymer microspheres by encapsulating modified graphene oxide nanosheets. Eur. Polym. J..

[12] Xu X., Bizmark N., Christie K.S.S., Datta S.S., Ren Z.J., Priestley R.D. (2022). Thermoresponsive Polymers for Water Treatment and Collection. Macromolecules.

[13] Guo Y., Guan W., Lei C., Lu H., Shi W., Yu G. (2022). Scalable super hygroscopic polymer films for sustainable moisture harvesting in arid environments. Nat. Commun..

[14] Baldwin P.M., Adler J., Davies M.C., Melia C.D. (1994). Holes in Starch Granules: Confocal, SEM and Light Microscopy Studies of Starch Granule Structure. Starch Stärke.

[15] Eichhorn S.J., Sampson W.W. (2010). Relationships between specific surface area and pore size in electrospun polymer fibre networks. J. R. Soc. Interface.

[16] Shanti N.O., Chan V.W.L., Stock S.R., De Carlo F., Thornton K., Faber K.T. (2014). X-ray micro-computed tomography and tortuosity calculations of percolating pore networks. Acta Mater..

[17] Sing K. (2001). The use of nitrogen adsorption for the characterization of porous materials. Colloids Surf. A Physicochem. Eng. Aspects.

[18] Determination of the Specific Surface Area of Solids by Gas Adsorption—BET Method.

[19] Detsi E. (2012). Metallic Muscles: Enhanced Strain and Electrolyte-Free Actuation. S.N. https://research.rug.nl/en/publications/metallic-muscles-enhanced-strain-and-electrolyte-free-actuation.

[20] Włodarczyk-Stasiak M., Jamroz J. (2009). Specific surface area and porosity of starch extrudates determined from nitrogen adsorption data. J. Food Eng..

[21] Sjostedt A. (2014). Preparation and Characterization of Nanoporous Cellulose Fibres and their Use in New Material Concepts. Doctoral Thesis.

[22] Ocieczek A., Mesinger D., Toczek H. (2022). Hygroscopic Properties of Three Cassava (Manihot esculenta Crantz) Starch Products: Application of BET and GAB Models. Foods.

[23] Smagin A.V., Sadovnikova N.B., Belyaeva E.A. (2022). Hygroscopicity of gel-forming composite materials: Thermodynamic assessment and technological significance. J. Compos. Sci..

[24] Andrade R.D., Lemus R., Perez C.E. (2011). Models of sorption isotherms for food: Uses and limitations. Vitae.

[25] Asomaning J.M., Sacande M., Olympio N.S. (2011). Water sorption isotherm characteristics of seeds of six indigenous forest tree species in Ghana. West Afr. J. Appl. Ecol..

[26] Ayala A.A., Serna-Cock L., Giraldo C.J.G. (2011). Moisture adsorption isotherms in yellow pitahaya (*Selenicereusmegalanthus*). Dyna.

[27] Amankwah E.A., Dsizi K.A., van Straten G., van Boxtel A.J.B. (2018). Modeling the equilibrium moisture content of desorption and adsorption of yam (Dente). Agric. Eng. Int. CIGR J..

[28] Resurreccion A.C., Moldrup P., Tuller M., Ferre T.P.A., Kawamoto K., Komatsu T., De Jonge L.W. (2011). Relationship between specific surface area and the dry end of the water retention curve for soils with varying clay and organic carbon contents. Water Resour. Res..

[29] Schneider M., Goss K.U. (2012). Prediction of the water sorption isotherm in air dry soils. Geoderma.

[30] Yan F., Tuller M., de Jonge L.W., Moldrup P., Arthur E. (2023). Specific surface area of soils with different clay mineralogy can be estimated from a single hygroscopic water content. Geoderma.

[31] Han S., Hong J., Luo Q., Xu H., Tan H., Wang Q., Tao J., Zhou Y., Peng L., He Y. (2022). Hygroscopicity of organic compounds as a function of organic functionality, water solubility, molecular weight, and oxidation level. Atmos. Chem. Phys..

[32] Carslaw K.S. (2022). Aerosols and Climate.

[33] Chan M.N., Choi M.Y., Ng N.L., Chan C.K. (2005). Hygroscopicity of water-soluble organic compounds in atmospheric aerosols: Amino acids and biomass burning derived organic species. Environ. Sci. Technol..

[34] Cheng Y., Su H., Koop T., Mikhailov E., Poschl U. (2015). Size dependence of phase transitions in aerosol nanoparticles. Nat. Commun..

[35] Deng Y., Kagami S., Ogawa S., Kawana K., Nakayama T., Kubodera R., Adachi K., Hussein T., Miyazaki Y., Mochida M. (2018). Hygroscopicity of Organic Aerosols and Their Contributions to CCN Concentrations Over a Midlatitude Forest in Japan. J. Geophys. Res. Atmos..

[36] Arigo A., Jawaha N., Nikhitha K., Jubie S. (2019). Effect of Hygroscopicity on pharmaceutical ingredients, methods to determine and overcome: An Overview. J. Pharm. Sci. Res..

[37] Tang M., Chan C.K., Li Y.J., Su H., Ma Q., Wu Z., Zhang G., Wang Z., Ge M., Hu M. (2019). A review of experimental techniques for aerosol hygroscopicity studies. Atmos. Chem. Phys..

[38] Acros Organics BV—Geel, Belgium. https://www.chemeurope.com/en/companies/12696/acros-organics-bv.html.

[39] SNF Water Science. https://www.snf.com.

[40] UPL. https://www.upl-ltd.com.

[41] Smagin A.V., Budnikov V.I., Sadovnikova N.B., Kirichenko A.V., Belyaeva E.A., Krivtsova V.N. (2022). Gel-Forming Soil Conditioners of Combined Action: Laboratory Tests for Functionality and Stability. Polymers.

[42] Arthur E., Tuller M., Moldrup P., de Jonge L.W. (2014). Evaluation of a Fully Automated Analyzer for Rapid Measurement of Water Vapor Sorption Isotherms for Applications in Soil Science. Soil Sci. Soc. Am. J..

[43] Smagin A.V. (2016). Thermogravimetric Determination of Specific Surface Area for Soil Colloids. Colloid J..

[44] Ioelovich M. (2021). Study of Hydrophilic Properties of Polysaccharides. Org. Polym. Mater. Res..

[45] Li R., Shi Y., Alsaedi M., Wu M., Shi L., Wang P. (2018). Hybrid hydrogel with high water vapor harvesting capacity for deployable solar-driven atmospheric water generator. Environ. Sci. Technol..

[46] Zhao F., Zhou X., Liu Y., Shi Y., Dai Y., Yu G. (2019). Super moisture-absorbent gels for all-weather atmospheric water harvesting. Adv. Mater..

[47] Diaz-Marin C.D., Zhang L., Lu Z., Alshrah M., Grossman J.C., Wang E.N. (2022). Kinetics of sorption in hygroscopic hydrogels. Nano Lett..

[48] Entezari A., Ejeian M., Wang R. (2020). Super Atmospheric Water Harvesting Hydrogel with Alginate Chains Modified with Binary Salts. ACS Mater. Lett..

[49] Wu M., Li R., Shi Y., Altunkaya M., Aleid S., Zhang C., Wang P. (2021). Metal and halide-free, solid-state polymeric water vapor sorbents for efficient water-sorption-driven cooling and atmospheric water harvesting. Mater. Horiz..

[50] Aleid S., Wu M., Li R., Wang W., Zhang C., Zhang L., Wang P. (2022). Salting-in effect of zwitterionic polymer hydrogel facilitates atmospheric water harvesting. ACS Mater. Lett..

[51] Graeber G., Diaz-Marin C.D., Gaugler L.C., Zhong Y., Fil B.E., Liu X., Wang E.N. (2023). Extreme Water Uptake of Hygroscopic Hydrogels through Maximized Swelling-Induced Salt Loading. Adv. Mater..

[52] Carotenuto A., Dell’Isola M. (1996). An experimental verification of saturated salt solution-based humidity fixed points. Int. J. Thermophys..

[53] Marestoni L.D., Wong A., Feliciano G.T., Marchi M.R.R., Tarley C.R.T., Sotomayor M.D.P.T. (2016). Semi-Empirical Quantum Chemistry Method for Pre-Polymerization Rational Design of Ciprofloxacin Imprinted Polymer and Adsorption Studies. J. Braz. Chem. Soc..

[54] Wuddivira M.N., Robinson D.A., Lebron I., Br´echet L., Atwell M., De Caires S., Oatham M., Jones S.B., Abdu H., Verma A.K. (2012). Estimation of soil clay content from hygroscopic water content measurements. Soil Sci. Soc. Am. J..

